# SLAM Project - Long Term Ecological Study of the Impacts of Climate Change in the Natural Forest of Azores: III - Testing the impact of edge effects in a native forest of Terceira Island

**DOI:** 10.3897/BDJ.10.e85971

**Published:** 2022-06-16

**Authors:** Paulo A. V. Borges, Lucas Lamelas-López, Noelline Tsafack, Mário Boieiro, Alejandra Ros-Prieto, Rosalina Gabriel, Rui Nunes, Maria Teresa Ferreira

**Affiliations:** 1 cE3c - Centre for Ecology, Evolution and Environmental Changes / Azorean Biodiversity Group & CHANGE – Global Change and Sustainability Institute, University of the Azores, Faculty of Agricultural Sciences and Environment, Rua Capitão João D` Ávila, São Pedro, 9700-042, Angra do Heroísmo, Azores, Portugal cE3c - Centre for Ecology, Evolution and Environmental Changes / Azorean Biodiversity Group & CHANGE – Global Change and Sustainability Institute, University of the Azores, Faculty of Agricultural Sciences and Environment, Rua Capitão João D` Ávila, São Pedro, 9700-042 Angra do Heroísmo, Azores Portugal; 2 IUCN SSC Mid-Atlantic Islands Specialist Group, Angra do Heroísmo, Azores, Portugal IUCN SSC Mid-Atlantic Islands Specialist Group Angra do Heroísmo, Azores Portugal; 3 Regional Secretariat of Environment and Climate Change, Project LIFE BEETLES (LIFE 18 NAT/PT/000864), Rua do Galo n. 118, 9700-040, Angra do Heroísmo, Azores, Portugal Regional Secretariat of Environment and Climate Change, Project LIFE BEETLES (LIFE 18 NAT/PT/000864), Rua do Galo n. 118, 9700-040 Angra do Heroísmo, Azores Portugal

**Keywords:** Arthropoda, Azores, edge effect, inventory, Macaronesia, temporal variation

## Abstract

**Background:**

The data we present are part of the long-term project “SLAM Project - Long Term Ecological Study of the Impacts of Climate Change in the Natural Forest of Azores” that started in 2012, aiming to understand the impact of biodiversity erosion drivers on Azorean native forests (Azores, Macaronesia, Portugal). The data for the current study consist in an inventory of arthropods collected in three locations of a native forest fragment at Terra-Brava protected area (Terceira, Azores, Portugal) aiming to test the impact of edge effects on Azorean arthropod communities. The three locations were: (i) the edge of the forest, closer to the pastures; (ii) an intermediate area (100 m from edge); and (iii) the deepest part of the native forest fragment (more than 300 m from edge). The study was carried out between June 2014 and December 2015. A total of nine passive flight interception SLAM (Sea, Land and Air Malaise) traps were deployed (three in each of the studied locations), during 18 consecutive months. This study provides the raw data to investigate temporal and edge effect variation for the Azorean arthropod communities.

**New information:**

The collected arthropods belong to a wide diversity of taxonomic groups of Arachnida, Diplopoda, Chilopoda and Insecta classes. We collected a total of 13,516 specimens from which it was possible to identify to species level almost all specimens (13,504). These identified specimens belong to 15 orders, 58 families (plus three with only genus or family level identification) and 97 species of arthropods. A total of 35 species are considered introduced, 34 native non-endemic and 28 endemic. Additionally, a total of 10 taxa (12 specimens) were recorded at genus, family or order level. This dataset will allow researchers to test the impact of edge effect on arthropod biodiversity and to investigate seasonal changes in Azorean arthropod native forest communities.

## Introduction

Arthropods are being affected by dramatic population declines and species extinctions worldwide (Sánchez-Bayo and Wyckhuys 2019, Cardoso et al. 2020, Harvey et al. 2020). One of the major causes for this biodiversity loss is the habitat destruction and degradation associated with replacement of native forest areas by other habitats, such as forest timber plantations, pasturelands etc. (e.g. Raven and Wagner 2021). These land use changes lead to habitat fragmentation by creating several isolated native forest patches of different size and morphology, thus leading to the exposure of forest-adapted biota to the conditions of the surrounding habitats, which is known as the “edge effect” (Murcia 1995). This interaction between two adjacent habitats (native forest and newly human-created habitat in this case) may have detrimental effects on the forest-adapted biotas due to changes in the biotic and abiotic conditions, mainly at the edges of the forest patches.

Oceanic islands have been especially affected by habitat degradation, as consequence of human colonisation (Triantis et al. 2010, Borges et al. 2019a, Borges et al. 2019b). In the Azores, the native habitats of the islands were strongly modified since Portuguese settlement starting in the 15^th^ century, by replacing native and pristine forests with pasturelands, agricultural areas, exotic tree plantations (e.g. *Cryptomeriajaponica* and *Eucalyptus* spp.) and urban areas. These major land-use changes actuated gradually in a gradient of elevation promoting the extinction of large-bodied beetle species (Terzopoulou et al. 2015) and the an ongoing process of extinction debt for many more (Triantis et al. 2010). Currently, the original forests comprise only about 5% of the total surface of the islands and are restricted to the most inaccessible areas (Gaspar et al. 2008, Triantis et al. 2010, Rego et al. 2015, Norder et al. 2020).

Some studies revealed a higher species richness and abundance of arthropods in the forest edges, which could have implications on the re-colonisation of adjacent altered habitats (Jokimäki et al. 1998, Magura 2002). Studies about Azorean arthropods have shown that the endemic species are mainly restricted to native forests and introduced species are more frequently detected in anthropogenic habitats, given their higher adaptability to the conditions of the newly-created habitat (Cardoso et al. 2009, Florencio et al. 2015, Florencio et al. 2016). The impact of invasive plants species is the major threat to endemic and native species in the native forest areas (Borges et al. 2019b) and their deleterious effects are probably more severe at the edges of the forests patches (Borges et al. 2006).

This publication is the seventh of a long-term monitoring project that started in the Azores in 2012 (SLAM Project - Long Term Ecological Study of the Impacts of Climate Change in the Natural Forest of Azores). The project was described in detail in Costa and Borges (2021) as a data-paper, but the first outputs were published earlier to test several ecological questions (see Borges et al. 2017, Matthews et al. 2018, Borges et al. 2020, de Vries et al. 2021). More recentlly, another data-paper was published investigating the occurrence of exotic and endemic arthropods in exotic and mixed forests and small disturbed remnants of native forests (Borges et al. 2022).

## General description

### Purpose

The current study is the third data-paper of the series and provides data from arthropod communities from Terra-Brava pristine native forest fragment (Terceira Island, Azores, Portugal) that will be useful to investigate: i) the impact of edge effects on biodiversity of arthropod communities from Terra-Brava pristine native forest and ii) seasonal changes in arthropod species richness and composition. In addition, the use of three replicate SLAM traps per (micro)habitat will be important to assess sampling completeness, perform sensitivity analyses and to support a cost-effective sampling design.

### Additional information

The data we present are part of the long-term project “SLAM Project - Long Term Ecological Study of the Impacts of Climate Change in the Natural Forest of Azores” that started in 2012, aiming to understand the impact of biodiversity erosion drivers on Azorean native forests (Azores, Macaronesia, Portugal). The current study includes new sampling areas in the native forests of Terceira Island and contributes with novel data that will be of paramount importance to obtain information about the native communities of arthropods across gradients of temporal and edge effects variation. Additionally, the samples collected in the most pristine areas contributed to the publication of Matthews et al. (2018) in testing the capacity of one single trap to capture relevant ecological properties (e.g. species composition, distribution of abundance) of the sampled communities. In two previous data papers, the project was described in detail and spider data were provided for several plots in Terceira and Pico Islands (Costa and Borges 2021) and the occurrence of exotic and endemic arthropods in exotic and mixed forests and small disturbed remnants of native forests was also investigated (Borges et al. 2022).

## Project description

### Title

SLAM Project III - Testing the impact of edge effects in native forests

### Personnel

The project was conceived and led by Paulo A.V. Borges.

Fieldwork: Paulo A. V. Borges, Rui Nunes.

Parataxonomists: Alejandra Ros-Prieto, David Rodilla Rivas, Juan Ignacio Pitarch Peréz, Laura Cáceres Sabater, Laura Gallardo, Marija Tomašić, Percy de Laminne de Bex, Rui Carvalho, William Razey.

Taxonomists: Paulo A. V. Borges.

Voucher specimen management was mainly undertaken by Alejandra Ros-Prieto and Paulo A. V. Borges.

### Study area description

**Figure 1.**
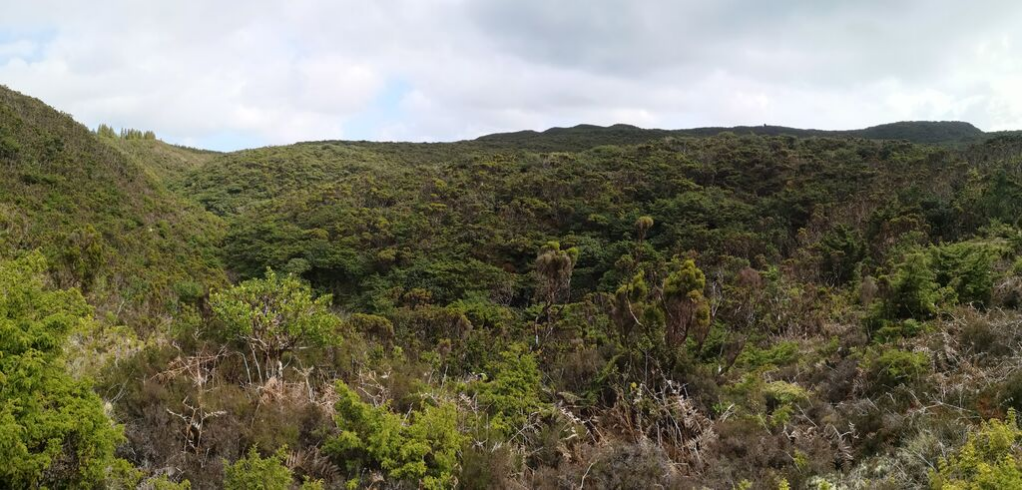
Terra-Brava Forest fragment on Terceira Island (Azores, Portugal) (Credit: Paulo A. V. Borges).

**Figure 2.**
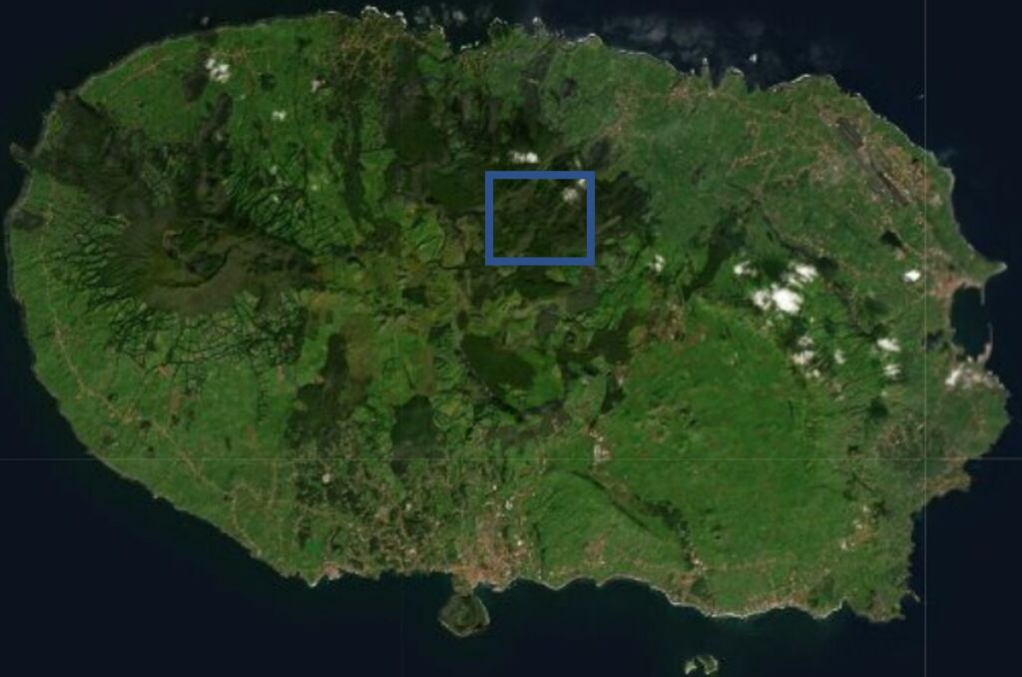
Location of Terra-Brava within Terceira Island, Azores (Credit: Paulo A. V. Borges).

**Figure 3.**
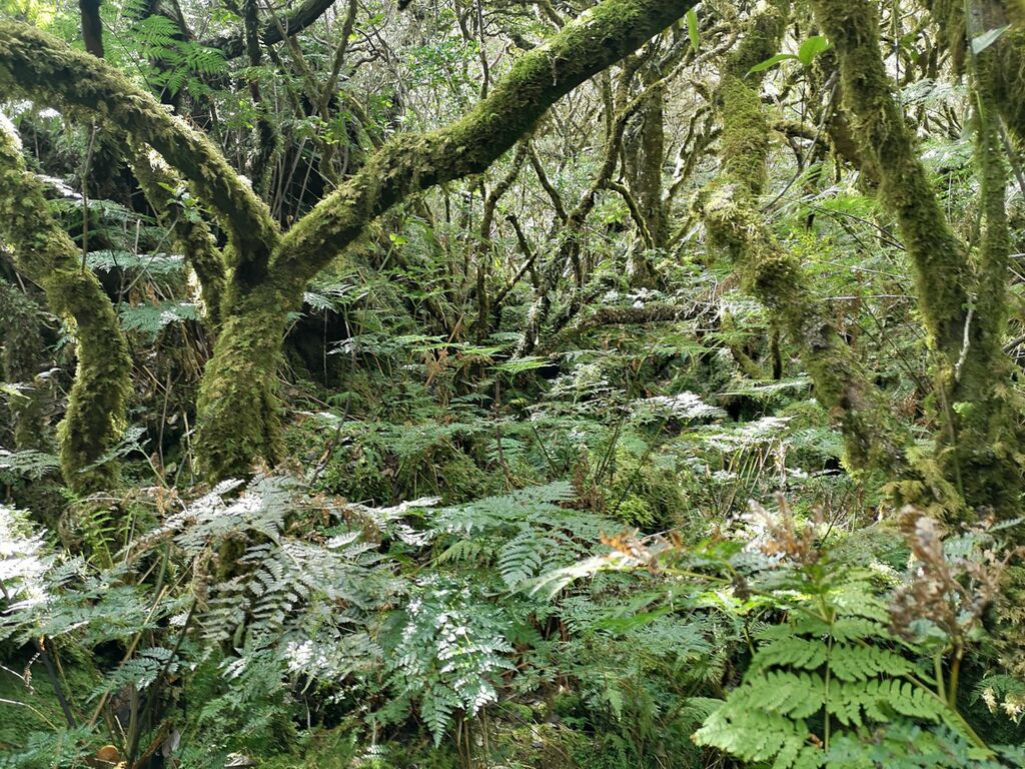
Terra-Brava dense carpet of bryophytes and ferns (Credit: Paulo A. V. Borges).

The study area comprises a fragment of the native forest “Terra-Brava” (Fig. 1) with an area of 180 ha located in the interior of Terceira Island (coordinates: 38°43'17"; -27°13'14") (Fig. 2), in the Azores Archipelago. The elevation ranges between 600 and 780 m a.s.l. This native forest fragment is pristine and covered by native vegetation, dominated by three endemic trees: *Laurusazorica* (Seub.) Franco (Laurales, Lauraceae), *Ilexazorica* Gand. (Aquifoliales, Aquifoliaceae) and *Juniperusbrevifolia* (Hochst. ex Seub.) Antoine (Pinales, Cupressaceae). The forest type of this forest fragment was classified by Elias et al. (2016) as “*Juniperus*-*Ilex* montane forests” that, in addition to a dominance of *J.brevifolia* and *I.azorica*, are characterised also by the presence of *L.azorica* at high densities and a dense cover of bryophytes and ferns in all substrates (Fig. 3). The shrub layer is dominated by the endemic species *Myrsineretusa* Aiton (Ericales, Myrsinaceae) and *Vacciniumcylindraceum* Sm. (Ericales, Ericaceae).

In general, the climate of the Archipelago is temperate oceanic, with frequent and abundant precipitation, high high relative humidity and persistent winds, mainly during the winter and autumn seasons.

### Design description

**Figure 4.**
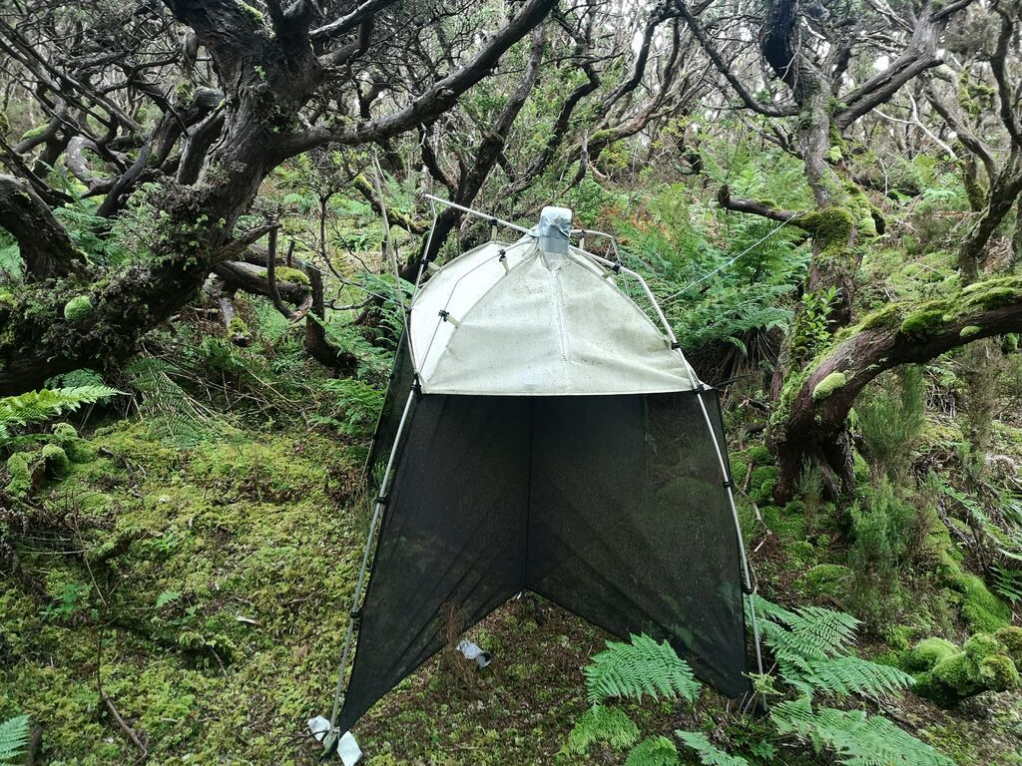
SLAM Trap (Sea, Land and Air Malaise trap) (Credit: Paulo A. V. Borges).

A total of nine SLAM (Sea, Land and Air Malaise) traps (Fig. 4) were deployed in the Terra-Brava native forest fragment: (i) three were set at the edge of the forest, closer to the pastures; (ii) three in an intermediate area (100 m from edge); and (iii) three in the deepest part of the native forest fragment (more than 300 m from edge) (Table 1). The trap samples were collected each month, during 18 consecutive months (from June 2014 to December 2015).

### Funding

A large number of students financed by the EU Programmes ERASMUS and EURODYSSÉE sorted the samples prior to species assignment. This manuscript was also partly financed by Portuguese FCT-NETBIOME –ISLANDBIODIV grant 0003/2011 (between 2012 and 2015), Portuguese National Funds, through FCT – Fundação para a Ciência e a Tecnologia, within the project UID/BIA/00329/2013-2020 and AZORESBIOPORTAL –PORBIOTA (ACORES-01-0145-FEDER-000072) (2019). The Natural Park of Terceira provided the necessary authorisation for arthropod sampling (Licence CCPI 006/2014). The database management and Open Access was funded by Fundação para a Ciência e a Tecnologia (FCT) through project “MACRISK-Trait-based prediction of extinction risk and invasiveness for Northern Macaronesian arthropods” - PTDC/BIA-CBI/0625/2021 (2022-2024).

## Sampling methods

### Sampling description

The data collection was performed using passive flight interception SLAM traps (Sea, Land and Air Malaise trap) (Fig. 4). Trap size is approximately 110 x 110 x 110 cm. The trap works on the principle that the intercepted arthropods crawl up the mesh and then fall inside the sampling recipient, which is filled with propylene glycol (pure 1,2-Propanodiol) (Borges et al. 2017). This sampling protocol is adequate to capture flying and non-flying arthropod species (Borges et al. 2017, Costa and Borges 2021) and it has been used to study diversity and abundance variations in the communities of arthropods on Azorean native areas (Matthews et al. 2018, Borges et al. 2020), to study patterns of diversity in a small elevation gradient (de Vries et al. 2021) and to investigate the role of exotic forests for the spread of exotic species and as a reservoir of relict populations of endemic species (Tsafack et al. 2021).

### Quality control

All sampled individuals were first sorted by trained paratoxonomists (see list above). All specimens were allocated to a taxonomic species by Paulo A. V. Borges. Juveniles are also included in the data presented in this paper since the low species diversity in the Azores allowed a relatively precise identification of this life-stage.

### Step description

At the laboratory, specimen sorting and arthropod identification followed standard procedures. A combination of somatic characters and reproductive structure was used for species identification. A reference collection was made for all collected specimens by assigning them a morphospecies code number and depositing them at the Dalberto Teixeira Pombo Insect Collection, University of Azores. Colonisation status for each identified species is based on Borges et al. (2010) (END -Endemic; NAT - native non-endemic; INT -introduced).

## Geographic coverage

### Description

Terra-Brava native forest fragment of Terceira Island, in the Azores Archipelago (Portugal).

### Coordinates

38°44'0.47'' and 38°48'50.4'' Latitude; 27°11'0.99''W and 27°13'20.66'' Longitude.

## Taxonomic coverage

### Description

The following Arthropod Classes and Orders are covered:

Arachnida: Araneae; Opiliones; Pseudoscorpiones.

Chilopoda: Lithobiomorpha.

Diplopoda: Chordeumatida, Julida.

Insecta: Archaeognatha; Blattodea; Coleoptera; Hemiptera; Neuroptera; Orthoptera; Psocodea; Thysanoptera; Trichoptera.

### Taxa included

**Table:** 

Rank	Scientific Name	Common Name
phylum	Arthropoda	Arthropods

## Temporal coverage

**Data range:** 2014-6-11 – 2015-12-14.

### Notes

Samples were taken monthly.

## Collection data

### Collection name

Entomoteca Dalberto Teixeira Pombo (DTP); University of Azores

### Collection identifier

DTP

### Specimen preservation method

All specimens were preserved in 96% ethanol.

### Curatorial unit

Curator: Paulo A. V. Borges

## Usage licence

### Usage licence

Creative Commons Public Domain Waiver (CC-Zero)

## Data resources

### Data package title

Monthly monitoring of Azorean forest arthropods testing for edge effects (Terceira Island, Azores, Portugal)

### Resource link

http://ipt.gbif.pt/ipt/resource?r=slam_edge&v=1.3

### Alternative identifiers

https://doi.org/10.15468/k84m4e

### Number of data sets

2

### Data set 1.

#### Data set name

Event Table

#### Data format

Darwin Core Archive format

#### Character set

UTF-8

#### Download URL

http://ipt.gbif.pt/ipt/resource?r=slam_edge&v=1.3

#### Data format version

Version 1.3

#### Description

The dataset was published in Global Biodiversity Information Facility platform, GBIF (Borges and Lamelas-López 2022). The following data table includes all the records for which a taxonomic identification of the species was possible. The dataset submitted to GBIF is structured as a sample event dataset that has been published as a Darwin Core Archive (DwCA), which is a standardised format for sharing biodiversity data as a set of one or more data tables. The core data file contains 158 records (eventID). This GBIF IPT (Integrated Publishing Toolkit, Version 2.5.6-rd6f172f) archives the data and thus serves as the data repository. The data and resource metadata are available for download in the Portuguese GBIF Portal IPT (Borges and Lamelas-López 2022).

**Data set 1.** 

Column label	Column description
eventID	Identifier of the events, unique for the dataset.
stateProvince	Name of the region of the sampling site.
islandGroup	Name of archipelago.
island	Name of the island.
country	Country of the sampling site.
countryCode	ISO code of the country of the sampling site.
municipality	Municipality of the sampling site.
decimalLatitude	The geographic latitude (in decimal degrees, using the spatial reference system given in geodeticDatum) of the geographic centre of a Location.
decimalLongitude	The geographic longitude (in decimal degrees, using the spatial reference system given in geodeticDatum) of the geographic centre of a Location.
geodeticDatum	The ellipsoid, geodetic datum or spatial reference system (SRS) upon which the geographic coordinates given in decimalLatitude and decimalLongitude are based.
coordinateUncertaintyInMetres	Uncertainty of the coordinates of the centre of the sampling plot in metres.
coordinatePrecision	A decimal representation of the precision of the coordinates given in the decimalLatitude and decimalLongitude.
georeferenceSources	A list (concatenated and separated) of maps, gazetteers or other resources used to georeference the Location, described specifically enough to allow anyone in the future to use the same resources.
locationID	Identifier of the location.
locality	Name of the locality.
locationRemarks	Additional information about the locality.
minimumElevationInMetres.	The lower limit of the range of elevation (altitude, usually above sea level), in metres.
habitat	The habitat of the sample.
year	Year of the event.
sampleSizeUnit	The unit of the sample size value.
eventDate	Date or date range the record was collected.
sampleSizeValue	The numeric amount of time spent in each sampling.
verbatimEventDate	The verbatim original representation of the date and time information for an Event. In this case, we use the season and year.
samplingProtocol	The sampling protocol used to capture the species.

### Data set 2.

#### Data set name

Occurrence Table

#### Data format

Darwin Core Archive format

#### Character set

UTF-8

#### Download URL

http://ipt.gbif.pt/ipt/resource?r=slam_edge&v=1.3

#### Data format version

Version 1.3

#### Description

The dataset was published in Global Biodiversity Information Facility platform, GBIF (Borges and Lamelas-López 2022). The following data table includes all the records for which a taxonomic identification of the species was possible. The dataset submitted to GBIF is structured as an occurrence table that has been published as a Darwin Core Archive (DwCA), which is a standardised format for sharing biodiversity data as a set of one or more data tables. The core data file contains 2779 records (occurrenceID). This GBIF IPT (Integrated Publishing Toolkit, Version 2.5.6-rd6f172f) archives the data and thus serves as the data repository. The data and resource metadata are available for download in the Portuguese GBIF Portal IPT (Borges and Lamelas-López 2022).

**Data set 2.** 

Column label	Column description
eventID	Identifier of the events, unique for the dataset.
type	Type of the record, as defined by the Public Core standard.
licence	Reference to the licence under which the record is published.
institutionID	The identity of the institution publishing the data.
institutionCode	The code of the institution publishing the data.
collectionID	The identity of the collection publishing the data.
collectionCode	The code of the collection where the specimens are conserved.
basisOfRecord	The nature of the data record.
occurrenceID	Identifier of the record, coded as a global unique identifier.
recordedBy	A list (concatenated and separated) of names of people, groups or organisations who performed the sampling in the field.
identifiedBy	A list (concatenated and separated) of names of people, groups or organisations who performed the sampling in the field.
dateIdentified	The date on which the subject was determined as representing the Taxon.
individualCount	A number or enumeration value for the quantity of organisms.
organismQuantityType	The type of quantification system used for the quantity of organisms.
lifeStage	The life stage of the organisms captured.
sex	The sex and quantity of the individuals captured.
scientificName	Complete scientific name including author and year.
scientificNameAuthorship	Name of the author of the lowest taxon rank included in the record.
kingdom	Kingdom name.
phylum	Phylum name.
class	Class name.
order	Order name.
family	Family name.
genus	Genus name.
specificEpithet	Specific epithet.
infraspecificEpithet	Infrapecific epithet.
taxonRank	Lowest taxonomic rank of the record.
establishmentMeans	The process of establishment of the species in the location, using a controlled vocabulary: in the GBIF database, we used the Borges et al. (2010) original classification: 'native', 'introduced', 'endemic'.
identificationRemarks	Information about morphospecies identification (code in Dalberto Teixeira Pombo Collection).

## Additional information

We collected a total of 13,516 specimens 13,504 of which were identified to species (Table 2). These identified specimens belong to 15 orders, 58 families (plus three with only genus or family level identification) and 97 species of arthropods. A total of 35 species are considered introduced, 34 native non-endemic and 28 endemic (Table 2). Additionally, a total of 10 taxa (12 specimens) were recorded at genus, family or order level (see Table 2).

Most species (S = 81) and specimens (n = 6588) were found in the traps located at greater distances from the edge (Table 2). Many species were also found in the edge areas (S = 77), including several exclusive (mostly introduced), but overall abundance was much lower in these areas.

The most abundant endemic species were the planthopper *Cixiusazoterceirae* Remane & Asche, 1979 (n = 2275), the spider *Rugathodesacoreensis* Wunderlich, 1992 (n = 1097) and the Archaeognatha jumping bristletail *Trigoniophthalmusborgesi* Mendes, Gaju, Bach & Molero, 2000 (n = 1046) (Table 2). The most abundant native non-endemic species were the harvestmen *Leiobunumblackwalli* Meade, 1861 (n = 1335), the aphid *Cinarajuniperi* (De Geer, 1773) (n = 715) and the flatid planthopper *Cyphopterumadcendens* (Herrich-Schäffer, 1835) (n = 687) (Table 2). The most abundant introduced species were the spider *Erofurcata* (Villers, 1789) (n = 207), the millipede *Ommatoiulusmoreletii* (Lucas, 1860) (n = 57) and the rove-beetle *Athetaaeneicollis* (Sharp, 1869) (n = 54) (Table 2).

**Figure 5.**
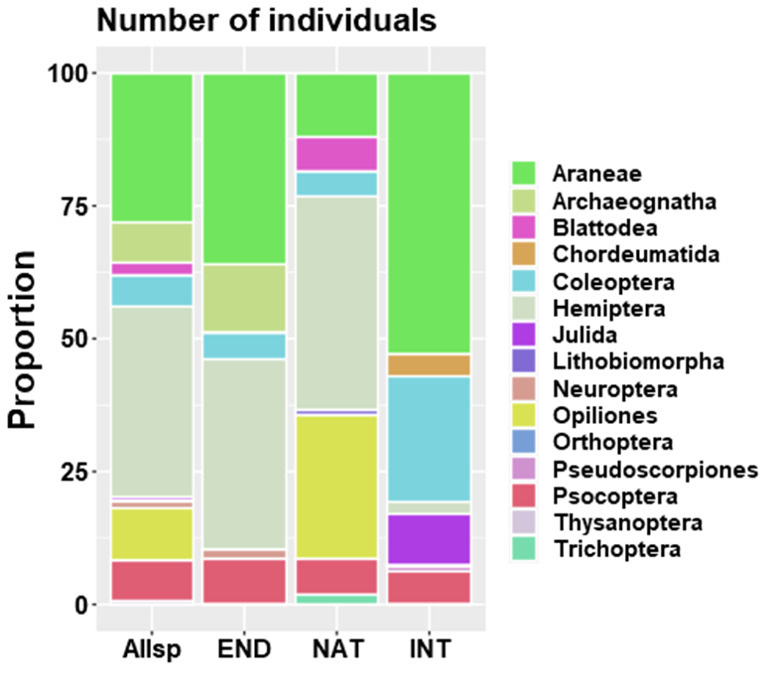
Proportional abundance of arthropods specimens (per order) sampled in the native forest fragment. Allsp = all arthropod species; END = endemic; NAT = native non-endemic; and INT = introduced.

Spiders (Araneae) and bugs (Hemiptera) dominate overall and endemic species abundance while Opiliones and Hemiptera include the most abundant non-endemic taxa (Fig. 5). Araneae and Coleoptera had the highest number of introduced specimens (Fig. 5).

**Figure 6.**
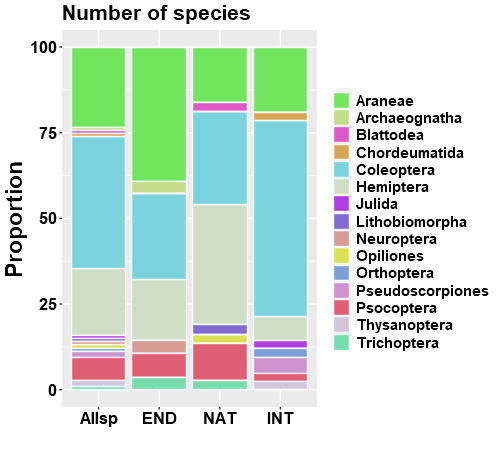
Proportional species richness of arthropods (per order) sampled in the native forest fragment. Allsp = all arthropod species; END = endemic; NAT = native non-endemic; and INT = introduced.

Proportionally, the most species-rich taxa are the beetles (Coleoptera), but spiders (Araneae) and bugs (Hemiptera) follow closely (Fig. 6). The same pattern applies when considering just the endemic and native non-endemic species, but Coleoptera are proportionally the most dominant taxon in the introduced species group (Fig. 6).

**Figure 7.**
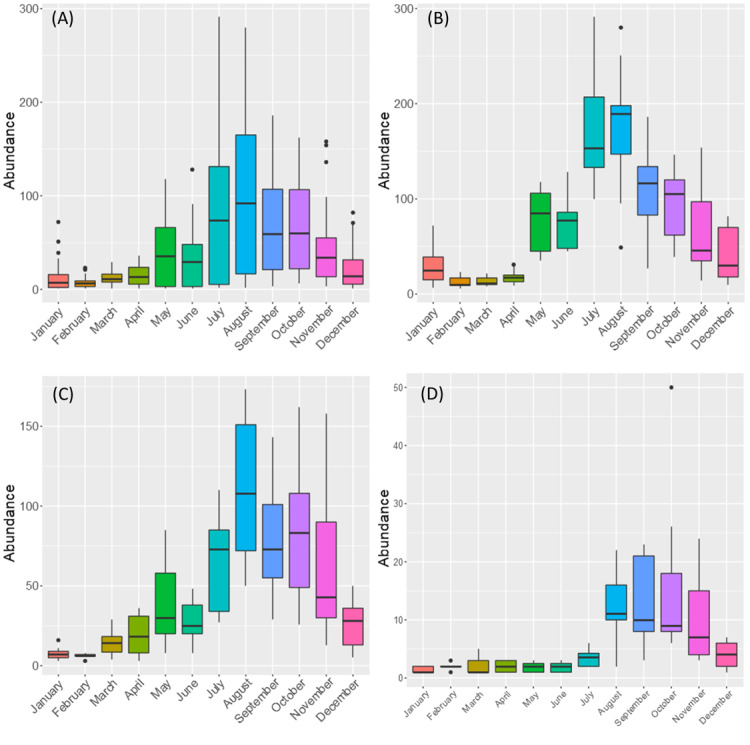
Boxplots of overall monthly variations of abundance for: (A) all species and separately for (B) endemic species, (C) native non-endemic species and (D) introduced species.

**Figure 8.**
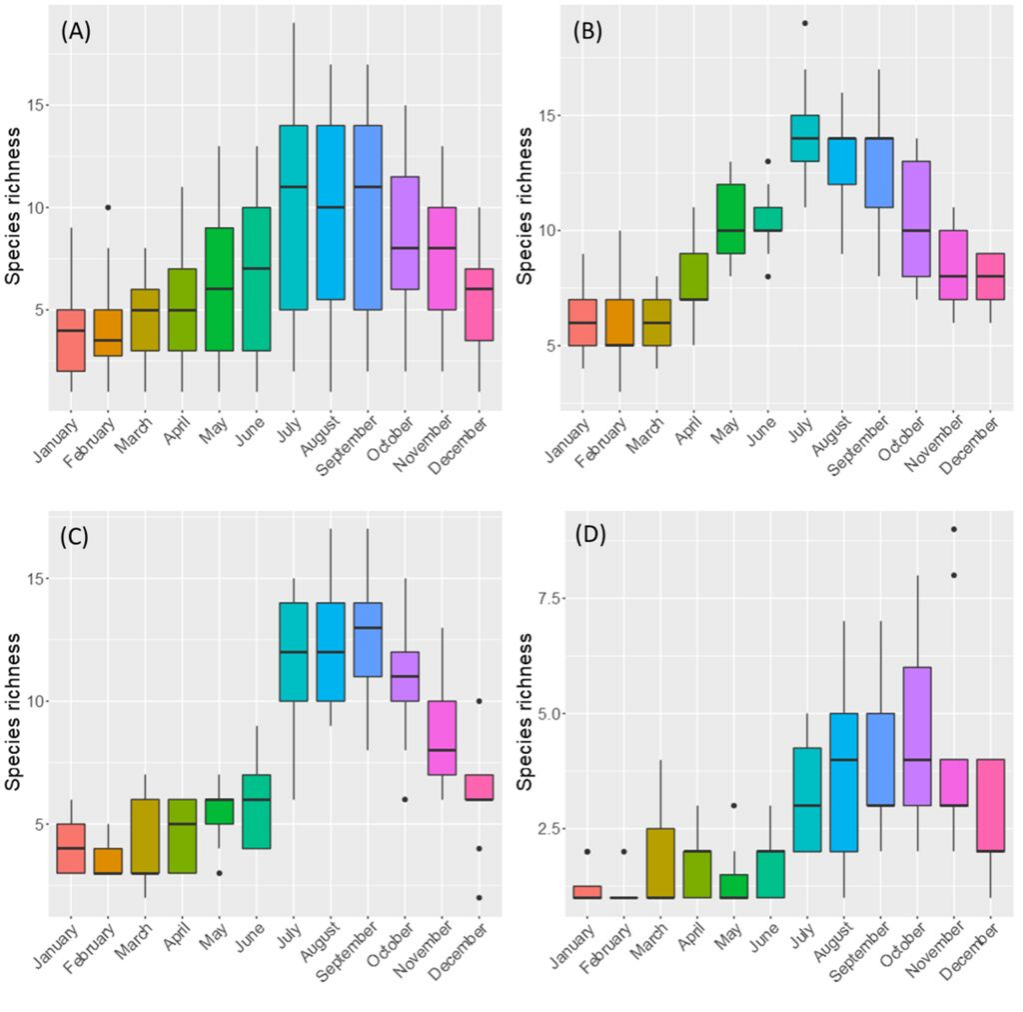
Boxplots of overall monthly variations of species richness for: (A) all species and separately for (B) endemic species, (C) native non-endemic species and (D) introduced species.

There are striking differences in specimen abundance and species richness throughout the sampling period (Figs 7, 8). The overall abundance of arthropod specimens presents a peak during July-October (unimodal) and this same pattern was found for endemic, native non-endemic and introduced groups of taxa (Fig. 7). Endemic arthropods were particularly abundant during July and August.

The variation in overall species richness also peaked during the summer months. The species richness patterns of the three groups of species (endemic, native-non-endemic and introduced) show a similar seasonal variation with very few species being active during winter and early spring (Fig. 8).

With this data, we are opening the possibility to investigate deeply the impact of edge effects in the Azorean hyper-humid native forests, which will be more accurately investigated in a classical research publication elsewhere. The scientific community interested in the use of SLAM traps for monitoring island forests have here also raw data to compare with other island systems (see also Borges et al. (2018) for best practices in monitoring island forest arthropods).

## Figures and Tables

**Table 1.** List of the nine sampled sites in the native forest fragment of “Terra-Brava”, in Terceira Island (Azores), between June 2014 and December 2015. Information is given about site location, site code, elevation (in metres) and decimal coordinates (Latitude and Longitude).

**Site location**	**Site code**	**Elevation**	**Latitude**	**Longitude**
Edge	TER-NFTB-T-18_Edge-A	650	38.73276	-27.19681
Edge	TER-NFTB-T-18_Edge-B	660	38.73232	-27.19657
Edge	TER-NFTB-T-18_Edge-C	660	38.73243	-27.19576
100 m inside	TER-NFTB-T-18_Original	670	38.73206	-27.1972
100 m inside	TER-NFTB-T-18_Centre	680	38.73235	-27.19798
100 m inside	TER-NFTB-T-18_Top	680	38.73272	-27.19827
300 m inside	TER-NFTB-T-18_Deep-A	680	38.7327	-27.20035
300 m inside	TER-NFTB-T-18_Deep-B	690	38.73227	-27.20012
300 m inside	TER-NFTB-T-18_Deep-C	700	38.73189	-27.19981

**Table 2.** Inventory of arthropod species collected in the native forest fragment of “Terra-Brava”, in Terceira Island (Azores), between June 2014 and December 2015. The list includes individuals identified at species-level and also morphospecies. Class, order, family, scientific name, morphospecies code (MF), colonisation status (CS: END – endemic; NAT - native non-endemic; INT – introduced;) and abundance per forest depth (i.e. at the edge of the forest - Edge, in the most pristine area - Deep and in an intermediate area between both - Centre) are provided.

**Class**	**Order**	**Family**	**MF**	**Scientific Name**	**CS**	**Edge**	**Centre**	**Deep**	**Tot**al
Arachnida	Araneae	Araneidae	134	*Gibbaraneaoccidentalis* Wunderlich, 1989	END	151	58	141	350
Arachnida	Araneae	Cheiracanthiidae	927	*Cheiracanthiumerraticum* (Walckenaer, 1802)	INT	1	0	2	3
Arachnida	Araneae	Clubionidae	516	*Porrhoclubionadecora* (Blackwall, 1859)	NAT	0	2	0	2
Arachnida	Araneae	Dictynidae	117	*Lathysdentichelis* (Simon, 1883)	NAT	96	72	92	260
Arachnida	Araneae	Dysderidae	28	*Dysderacrocata* C.L. Koch, 1838	INT	3	2	47	52
Arachnida	Araneae	Linyphiidae	2	*Tenuiphantesmiguelensis* (Wunderlich, 1992)	NAT	9	2	15	26
Arachnida	Araneae	Linyphiidae	4	*Porrhommaborgesi* Wunderlich, 2008	END	1	1	3	5
Arachnida	Araneae	Linyphiidae	21	*Tenuiphantestenuis* (Blackwall, 1852)	INT	28	2	12	42
Arachnida	Araneae	Linyphiidae	34	*Erigoneatra* Blackwall, 1833	INT	0	0	1	1
Arachnida	Araneae	Linyphiidae	50	*Canariphantesacoreensis* (Wunderlich, 1992)	END	6	0	3	9
Arachnida	Araneae	Linyphiidae	181	*Savigniorrhipisacoreensis* Wunderlich, 1992	END	213	211	598	1022
Arachnida	Araneae	Linyphiidae	233	*Oedothoraxfuscus* (Blackwall, 1834)	INT	2	0	0	2
Arachnida	Araneae	Linyphiidae	234	*Erigoneautumnalis* Emerton, 1882	INT	1	0	0	1
Arachnida	Araneae	Linyphiidae	246	*Erigonedentipalpis* (Wider, 1834)	INT	0	0	1	1
Arachnida	Araneae	Linyphiidae	312	*Acorigoneacoreensis* (Wunderlich, 1992)	END	29	70	56	155
Arachnida	Araneae	Linyphiidae	421	*Walckenaeriagrandis* (Wunderlich, 1992)	END	2	1	1	4
Arachnida	Araneae	Linyphiidae	442	*Miniciafloresensis* Wunderlich, 1992	END	0	23	21	44
Arachnida	Araneae	Linyphiidae	697	*Microlinyphiajohnsoni* (Blackwall, 1859)	NAT	114	38	107	259
Arachnida	Araneae	Lycosidae	17	*Pardosaacorensis* Simon, 1883	END	1	0	0	1
Arachnida	Araneae	Mimetidae	140	*Erofurcata* (Villers, 1789)	INT	47	76	84	207
Arachnida	Araneae	Pisauridae	39	*Pisauraacoreensis* Wunderlich, 1992	END	4	9	13	26
Arachnida	Araneae	Salticidae	198	*Macaroeriscata* (Blackwall, 1867)	NAT	12	9	23	44
Arachnida	Araneae	Tetragnathidae	179	*Sancusacoreensis* (Wunderlich, 1992)	END	80	24	64	168
Arachnida	Araneae	Theridiidae	5	*Rugathodesacoreensis* Wunderlich, 1992	END	114	519	464	1097
Arachnida	Araneae	Thomisidae	3	*Xysticuscor* Canestrini, 1873	NAT	0	4	1	5
Arachnida	Opiliones	Phalangiidae	6	*Leiobunumblackwalli* Meade, 1861	NAT	289	373	673	1335
Arachnida	Pseudoscorpiones	Chthoniidae	38	*Chthoniusischnocheles* (Hermann, 1804)	INT	0	0	2	2
Arachnida	Pseudoscorpiones	Neobisiidae	296	*Neobisiummaroccanum* Beier, 1930	INT	0	3	1	4
Chilopoda	Lithobiomorpha	Lithobiidae	27	*Lithobiuspilicornispilicornis* Newport, 1844	NAT	7	23	12	42
Diplopoda	Chordeumatida	Haplobainosomatidae	468	*Haplobainosomalusitanum* Verhoeff, 1900	INT	10	14	0	24
Diplopoda	Julida	Julidae	9	*Ommatoiulusmoreletii* (Lucas, 1860)	INT	25	3	29	57
Insecta	Archaeognatha	Machilidae	144	*Trigoniophthalmusborgesi* Mendes, Gaju, Bach & Molero, 2000	END	209	375	462	1046
Insecta	Blattodea	Corydiidae	59	*Zethasimonyi* (Krauss, 1892)	NAT	46	110	151	307
Insecta	Coleoptera	Carabidae	45	*Anisodactylusbinotatus* (Fabricius, 1787)	INT	1	0	0	1
Insecta	Coleoptera	Cerambycidae	147	*Crotchiellabrachyptera* Israelson, 1985	END	3	1	1	5
Insecta	Coleoptera	Chrysomelidae	266	*Chaetocnemahortensis* (Fourcroy, 1785)	INT	1	0	0	1
Insecta	Coleoptera	Chrysomelidae	395	*Psylliodesmarcida* (Illiger, 1807)	NAT	1	0	2	3
Insecta	Coleoptera	Chrysomelidae	679	Chrysomelidae	??		0	1	1
Insecta	Coleoptera	Chrysomelidae	1246	*Phylotreta*	INT	1	0	0	1
Insecta	Coleoptera	Ciidae	107	*Atlantocisgillerforsi* Israelson, 1985	END	10	0	2	12
Insecta	Coleoptera	Corylophidae	65	*Sericoderuslateralis* (Gyllenhal, 1827)	INT	0	0	1	1
Insecta	Coleoptera	Cryptophagidae	145	*Cryptophagus*	INT	0	0	2	2
Insecta	Coleoptera	Curculionidae	46	*Drouetiusborgesiborgesi* (Machado, 2009)	END	1	6	20	27
Insecta	Coleoptera	Curculionidae	102	*Pseudophloeophagustenaxborgesi* Stüben, 2022	NAT	21	20	65	106
Insecta	Coleoptera	Curculionidae	141	*Calacallessubcarinatus* (Israelson, 1984)	END	16	10	47	73
Insecta	Coleoptera	Curculionidae	237	*Xyleborinusalni* Nijima, 1909	INT	2	0	0	2
Insecta	Coleoptera	Curculionidae	344	*Sitonadiscoideus* Gyllenhal, 1834	INT	0	2	1	3
Insecta	Coleoptera	Curculionidae	568	*Phloeosinusgillerforsi* Bright, 1987	END	0	1	0	1
Insecta	Coleoptera	Curculionidae	673	*Mecinuspascuorum* (Gyllenhal, 1813)	INT	0	1	0	1
Insecta	Coleoptera	Dryopidae	286	*Dryopsalgiricus* (Lucas, 1846)	NAT	0	1	0	1
Insecta	Coleoptera	Elateridae	244	*Alestrusdolosus* (Crotch, 1867)	END	0	1	1	2
Insecta	Coleoptera	Hydrophilidae	40	*Cercyonhaemorrhoidalis* (Fabricius, 1775)	INT	6	2	2	10
Insecta	Coleoptera	Hydrophilidae	342	*Cercyon*	INT	1	0	0	1
Insecta	Coleoptera	Laemophloeidae	98	*Placonotus*	NAT	0	0	1	1
Insecta	Coleoptera	Laemophloeidae	110	*Cryptolestes*	NAT	0	1	0	1
Insecta	Coleoptera	Laemophloeidae	705	Laemophloeidae	INT	0	1	0	1
Insecta	Coleoptera	Latridiidae	710	*Cartoderenodifer* (Westwood, 1839)	INT	2	0	1	3
Insecta	Coleoptera	Latridiidae	733	*Cartoderebifasciata* (Reitter, 1877)	INT	0	0	1	1
Insecta	Coleoptera	Leiodidae	257	*Catopscoracinus* Kellner, 1846	NAT	6	3	25	34
Insecta	Coleoptera	Monotomidae	708	*Monotoma*	INT	0	0	2	2
Insecta	Coleoptera	Ptiliidae	72	*Ptenidiumpusillum* (Gyllenhal, 1808)	INT	1	0	0	1
Insecta	Coleoptera	Scraptiidae	78	*Anaspisproteus* Wollaston, 1854	NAT	21	32	20	73
Insecta	Coleoptera	Staphylinidae	16	*Athetafungi* (Gravenhorst, 1806)	INT	2	0	0	2
Insecta	Coleoptera	Staphylinidae	41	*Ocypusaethiops* (Waltl, 1835)	NAT	1	2	12	15
Insecta	Coleoptera	Staphylinidae	52	*Cordaliaobscura* (Gravenhorst, 1802)	INT	1	0	0	1
Insecta	Coleoptera	Staphylinidae	57	*Athetaaeneicollis* (Sharp, 1869)	INT	38	6	10	54
Insecta	Coleoptera	Staphylinidae	79	*Quediuscurtipennis* Bernhauer, 1908	NAT	0	0	3	3
Insecta	Coleoptera	Staphylinidae	82	*Proteinusatomarius* Erichson, 1840	NAT	0	1	2	3
Insecta	Coleoptera	Staphylinidae	89	*Tachyporusnitidulus* (Fabricius, 1781)	INT	2	2	7	11
Insecta	Coleoptera	Staphylinidae	142	*Tachyporuschrysomelinus* (Linnaeus, 1758)	INT	1	1	2	4
Insecta	Coleoptera	Staphylinidae	247	*Aleocharabipustulata* (Linnaeus, 1760)	INT	2	1	1	4
Insecta	Coleoptera	Staphylinidae	265	*Xantholinuslongiventris* Heer, 1839	INT	1	2	1	4
Insecta	Coleoptera	Staphylinidae	439	*Notothectadryochares* (Israelson, 1985)	END	27	22	213	262
Insecta	Coleoptera	Staphylinidae	825	*Athetaatramentaria* (Gyllenhal, 1810)	INT	23	0	3	26
Insecta	Hemiptera	Anthocoridae	521	*Brachystelesparvicornis* (A. Costa, 1847)	NAT	0	0	1	1
Insecta	Hemiptera	Aphididae	60	*Rhopalosiphoninuslatysiphon* (Davidson, 1912)	INT	3	1	0	4
Insecta	Hemiptera	Cicadellidae	8	*Aphrodeshamiltoni* Quartau & Borges, 2003	END	0	0	1	1
Insecta	Hemiptera	Cicadellidae	465	*Eupteryxazorica* Ribaut, 1941	END	7	1	1	9
Insecta	Hemiptera	Cicadellidae	1019	*Eupteryxfilicum* (Newman, 1853)	NAT	1	1	0	2
Insecta	Hemiptera	Cicadellidae	1021	Cicadellidae		1	0	0	1
Insecta	Hemiptera	Cixiidae	7	*Cixiusazoterceirae* Remane & Asche, 1979	END	469	663	1143	2275
Insecta	Hemiptera	Corixidae	1039	*Corixaaffinis* Leach, 1817	NAT	0	0	1	1
Insecta	Hemiptera	Delphacidae	254	*Megamelodesquadrimaculatus* (Signoret, 1865)	NAT	0	0	7	7
Insecta	Hemiptera	Delphacidae	321	*Kelisiaribauti* Wagner, 1938	NAT	0	1	0	1
Insecta	Hemiptera	Delphacidae	1252	Delphacidae	INT	0	0	1	1
Insecta	Hemiptera	Flatidae	124	*Cyphopterumadcendens* (Herrich-Schäffer, 1835)	NAT	187	135	365	687
Insecta	Hemiptera	Lachnidae	44	*Cinarajuniperi* (De Geer, 1773)	NAT	164	75	476	715
Insecta	Hemiptera	Lygaeidae	167	*Kleidocerysericae* (Horváth, 1908)	NAT	1	5	12	18
Insecta	Hemiptera	Miridae	137	*Pinalitusoromii* J. Ribes, 1992	END	81	186	296	563
Insecta	Hemiptera	Miridae	476	*Monalocorisfilicis* (Linnaeus, 1758)	NAT	18	0	2	20
Insecta	Hemiptera	Miridae	1137	*Trigonotyluscaelestialium* (Kirkaldy, 1902)	NAT	0	1	0	1
Insecta	Hemiptera	Nabidae	230	*Nabispseudoferusibericus* Remane, 1962	NAT	1	1	6	8
Insecta	Hemiptera	Psyllidae	557	*Strophingiaharteni* Hodkinson, 1981	END	10	25	10	45
Insecta	Hemiptera	Psyllidae	662	*Acizziauncatoides* (Ferris & Klyver, 1932)	INT	5	1	2	8
Insecta	Hemiptera	Triozidae	195	*Triozalaurisilvae* Hodkinson, 1990	NAT	261	74	174	509
Insecta	Neuroptera	Hemerobiidae	200	*Hemerobiusazoricus* Tjeder, 1948	END	33	33	85	151
Insecta	Orthoptera	Gryllidae	245	*Eumodicogryllusbordigalensis* (Latreille, 1804)	INT	1	0	0	1
Insecta	Psocodea	Caeciliusidae	191	*Valenzuelaflavidus* (Stephens, 1836)	NAT	41	46	80	167
Insecta	Psocodea	Caeciliusidae	625	*Valenzuelaburmeisteri* (Brauer, 1876)	NAT	1	0	0	1
Insecta	Psocodea	Ectopsocidae	121	*Ectopsocusbriggsi* McLachlan, 1899	INT	9	8	18	35
Insecta	Psocodea	Elipsocidae	184	*Elipsocusazoricus* Meinander, 1975	END	53	3	21	77
Insecta	Psocodea	Elipsocidae	370	*Elipsocusbrincki* Badonnel, 1963	END	224	147	224	595
Insecta	Psocodea	Epipsocidae	374	*Bertkauialucifuga* (Rambur, 1842)	NAT	50	15	42	107
Insecta	Psocodea	Trichopsocidae	478	*Trichopsocusclarus* (Banks, 1908)	NAT	28	8	16	52
Insecta	Thysanoptera	Phlaeothripidae	13	*Hoplothripscorticis* (De Geer, 1773)	NAT	7	6	75	88
Insecta	Thysanoptera	Thripidae	280	*Hercinothripsbicinctus* (Bagnall, 1919)	INT	0	0	1	1
Insecta	Trichoptera	Limnephilidae	432	*Limnephilusatlanticus* Nybom, 1948	END	1	0	0	1
**Grand Total**						**3349**	**3579**	**6588**	**13516**
